# A chronicle of the changes undergone by a maritime territory, the Bay of Toulon (Var Coast, France), and their consequences on PCB contamination

**DOI:** 10.1186/s40064-016-2715-2

**Published:** 2016-08-02

**Authors:** Emmanuel Wafo, Lydia Abou, Alain Nicolay, Pierre Boissery, Thierry Perez, Rose Ngono Abondo, Cédric Garnier, Mama Chacha, Henri Portugal

**Affiliations:** 1 Laboratoire de Chimie Analytique, IMBE UMR 7263 CNRS, 237IRD/l’UMR 1062 INSERM/INRA 1260/AMU- NORT: Nutrition, Obésité et Risques Thrombotique et UMR 910 Génétique, Marseille, France; 2 Aix-Marseille-Université, Laboratoire de Chimie Analytique associé à l’UMR 1062 INSERM/INRA 1260/AMU-NORT: Nutrition, Obésité et Risques Thrombotique et UMR 910 Génétique, Marseille, France; 3 Aix-Marseille-Université, Agence de l’Eau Rhône Méditerranée Corse, 62, La Canebière, Marseille, France; 4 IMBE UMR CNRS 7263/IRD 237, Aix-Marseille-Université, Université d’Avignon, Avignon, France; 5 Laboratoire de Pharmacie Galénique et de Législation Pharmaceutique, Faculté de Médecine et des Sciences Biomédicales, Université de Yaoundé 1, Yaounde, Cameroon; 6 Laboratoire PROTEE, Université du Sud de Toulon, Toulon, France; 7 FEAS, Alhosn University, Abu Dhabi, United Arab Emirates

**Keywords:** Toulon Bay, Sediment, Polychlorinated biphenyls (PCBs), Reference values, Historicity, Depth profile

## Abstract

This study evaluated the distribution of polychlorinated biphenyls (PCBs) in 39 surface sediment samples and four cores collected in Toulon Bay, a semiclosed area submitted to various anthropogenic inputs. The concentration of PCBs in the superficial sediment samples ranged from 1.7 to 2530 ng g^−1^ dry weight. The spatial distribution of these compounds suggested that the high concentrations of these contaminants are located in the small bay and are related to human activities. In the larger bay, the concentrations were in the same order of magnitude than those reported in others locations around the world. Comparison of the levels with target values from the French legislation shows that, except for four polluted sites with critical values (N2: values ≥1 mg kg^−1^ dry weight) in the smaller bay, PCBs levels throughout the larger and the smaller bay are lower than the accepted values (N1: values <0.5 mg kg^−1^ dry weight). The PCBs in the sediment cores ranged from 0.8 to 739 ng g^−1^ dry weight dependent core. Vertical profiles indicated earlier usage of PCBs which coincided with the history of the Toulon Bay. In this study, using alkane, we could follow the PCBs pollution history over about 80 years and estimate a sedimentation rate of about 0.32 cm year in the small Bay of Toulon.

**Electronic supplementary material:**

The online version of this article (doi:10.1186/s40064-016-2715-2) contains supplementary material, which is available to authorized users.

## Backgrounds

Polychlorinated biphenyls (PCBs) were discovered in the early 1900 s. They form a large family of about 209 different ‘congener’ molecules that differ in the number of chlorine atoms (1–10 Cl) (Hutzinger et al. 1974; Hawker and Connell 1988), their locations in the biphenyl core and other spatial properties. From the 1920s onwards, PCBs have found multiple uses, due to their high thermodynamic stability and chemically inert nature (Martin et al. 2003; Fu and Wu 2006; Darko et al. 2008). However, because of their high stability, environmental PCBs degrade very slowly. Since these molecules are lipophilic, they concentrate in many biological organisms and exert toxic effects of long and unpredictable duration. PCBs can be stored in sediments over decades (Bellemin-Guyot 1982; Monod et al. 1987; De Voogt and Brinkman 1989; Duan et al. 2013b). This persistence varies among different congeners, and so the relative proportions of different molecules in a given medium vary in space and time according to physical, chemical and biological factors. (MacKay et al. 1992) reported that the half-life of PCBs ranges approximately from 8 months to 6 years depending on the congeners. Industrial processes allowing the decomposition of PCBs implements chemical and thermal technologies, which have no equivalent in the natural environment. The only one bacterial (Swers and May 2013; Chun et al. 2013) natural process may realize a slow biodegradation into two phases: the first one, anaerobic, being able to dechlorinate gradually PCBs. The second, aerobic, transfers the dechlorinated products towards the interface water–sediment.

Environmental risks depend on PCBs persistence in sediments and on their accumulation in organisms of the food chain up to the human consumer. The main effects are metabolic disorders, leading for instance to altered calcification and changes in reproductive capacity of marine species (Colborn and Smolen 1996; Xue et al. 2005). Even so, despite the accumulation of PCBs up to 450 mg kg^−1^ in the fatty tissues of marine mammals (Wafo, personal communication), the links with mortality have been difficult to establish (Hany et al. 1999).

PCB dispersion in a coastal environment is closely linked to particulate matter, including the dispersion, nature and supply of this particulate matter, its absorbent properties and movements according to coastal currents and to the depths of the seafloor.

Our study focuses on the evaluation of the level of PCBs contamination in the sediments of the Bay of Toulon. This bay is semi-enclosed, exposed to strong anthropic impact and to the dumping of a number of contaminants, leading to the progressive degradation of the environment. This is exemplified by the almost complete disappearance of the Posidonia beds (Bernard et al. 2001), testimony to a strong perturbation of the underwater biological habitat. Based on this observation, a “Bay Contract” was established for the Bay of Toulon by the “Syndicat Intercommunal de l’Aire Toulonnaise” (Toulon Area Intercommunal Association, SIAT. The aim is to restore healthy aquatic ecosystems, to preserve the heritage and enhance the economy.

Within the frame of this bay contract and on the basis of a prior diagnostic, the objectives of restoration of water quality and preservation of the marine environment were set up and the actions and works necessary to restore and valorise the water quality were planned. To meet these requirements, the CARTOCHIM project for the Toulon Bay was initiated in 2008. Among the actions to be undertaken, the determination of pollutants such as metals, polychlorinated biphenyl (PCBs), polycyclic aromatic hydrocarbons (PAHs), tributyltin (TBT) appeared as a priority. Since the early 1990s, PCBs levels from along most of the Mediterranean coast, including the Toulon Bay and its surroundings, have been studied in our laboratory both in surface sediment and along core samplings. Sediment can be seen as a geologic history or memory that retains patterns from the past.

The Toulon harbour has experienced a series of events that are likely to have played a role in affecting the sediment at different dates: battles, the sinking of ships, pier constructions, river diversions, and industrial developments. We can attempt to identify the traces of these events to estimate the sedimentation speed at different sites. Sediment deposition depend not only on the vertical accretion of minerals, organic and biological matter in water but also on their horizontal transfer mediated by sea-water currents, along with bioturbation by invertebrates and burrowing fishes. The speed of sedimentation depends not only on the origin of mineral and biological particles but also on the strengths of sea currents. This phenomenon is enhanced close to the shore where depths are low and topography plays a major role. The construction of the pier which divides the Toulon Bay into two areas complicates the problem. One part is heavily urbanised and industrialised, and the other is opened to the sea and is mostly devoted to touristic uses. In these conditions, settling velocities for pollutants may best be treated globally. Nevertheless, a partial approach to the problem in the smaller bay, and especially its southern zones, would be most useful since they receive pollution coming directly from the shore and the dockyards.

A study of alkanes, reported by Milano (1990), was made using a 60 cm deep sample obtained from a site very close to sample site 15 of this study. We used the raw data from this work to analyse the speed of pollutant deposition in the region and to estimate a chronology for PCBs pollution at the sites sampled in our study.

Thus, the aims of this study were to: (a) measure the level of contamination by PCBs of the sediments in the Bay of Toulon and estimate the sediment toxicity with respect to reference values; (b) study the chronology of this pollution and relate the temporal trend in pollutants concentrations to the history of the Toulon.

## Methods

### Study area

**Fig. 1:**
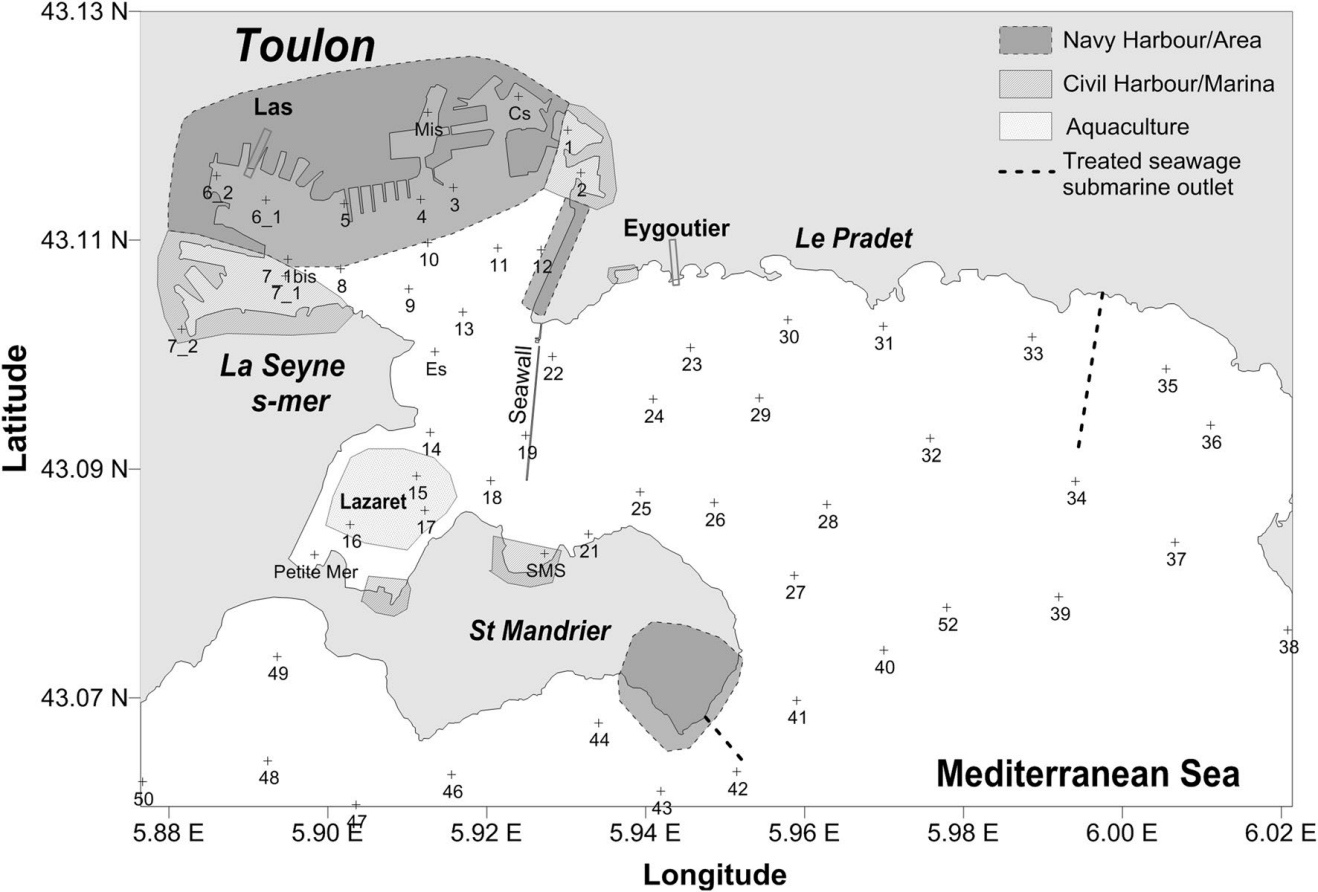
Map of the sampling sites in Toulon Bay

Toulon Bay covers a large area (Fig. 1) extending to the open sea between the point of Carqueiranne in the east and Cap Cepet in the west. It is protected from north winds by Mounts Caume, Coudon and Faron and from south winds by the layered crystalline rock formations of Carqueiranne in the east and St Mandrier in the west. Two natural bays are linked: the larger bay, with an area of 31 km^2^, shows a semi-circular shape, opens to the Mediterranean and offers little shelter from eastern winds. The smaller, western bay is much better protected by several promontories that provide shelter for harbours and docks and many military, industrial, tourist and aquaculture activities. At the end of the 19th century, a pier (1500 m) was built between Mourillon and St Mandrier isolating the two areas more.

Two rivers with relatively weak currents flow into the Toulon Bay basin: the Eygoutier (12 km) and the Las (8 km). These two rivers, which are subject to the Mediterranean tide, have always experienced severe and significant changes in water level. The Eygoutier has been diverted to the larger eastern bay to lessen this problem. The depth profile of the waters of Toulon Bay reveals two isobaths at depths of 50 and 60 m at less than 2 km from the head of the canyon that cuts the continental slope to the south and in the axis of the bay.

Differences in depths in the larger, eastern bay, mostly greater than 20 m, and the western bay, typically less than 20 m, were accentuated by the construction of the pier. Sedimentation in the coastal zone was enhanced especially in harbours and coves (Bregaillon and Lazaret) less than 10 m in depth. The channel between the two bays was reduced from 3 km to about 500 m by the construction of the pier. A deeper channel was dredged and is maintained artificially at a depth of 20 m. It runs along the peninsula of St Mandrier and takes a steeper slope towards the centre of the bay before joining the head of the canyon to the South.

As the expansion of Toulon town was closely linked to the expansion of the fleet and dockyards, military setbacks, including those of 1707, 1793 and 1942, have caused major losses in ground installations such as the dockyards, harbours, forts, and munition and fuel supplies. Major changes occurred after the Second World War. The naval strength remained but the shift to nuclear-powered aircraft-carriers and submarines altered the impact on the environment. Over the same years, commercial sea traffic greatly increased, and the democratization of sailing for pleasure has equally increased maritime traffic.

Further changes have occurred as the Toulon metropolis has grown to a population of nearly 600,000. It now extends from Ollioules in the west to Pradet in the east and from La Seyne to St Mandrier in the south. The conurbation has grown very fast, with large increases in industrial, commercial and tourist activities. Contributions from pollutants due to these activities have increased, with runoffs and outflows from impermeable surfaces. The sources have become more dispersed, resulting in a diffuse pollution widely distributed throughout Toulon Bay.

### Sampling and storage

Surface sediments were sampled in November 2008, February and June 2009, with the help of the French Navy. We collected samples of ‘superficial sediment’ by pulling 10 cm long cylindrical tubes into the sediment at 39 points distributed across the bay. Each sample was subdivided in two parts corresponding to depths of 0–5 and 5–10 cm. The sampling procedures and sample treatment had been described by (Tessier et al. 2011).

Four additional interface cores, representative of conditions over time for different sites in the bay (core 12 and 15 for the small bay; 23 and 52 for the larger bay), were collected in the same conditions, to evaluate the time course of PCBs contamination. They were divided into 2 cm slices under liquid nitrogen and stored at −20 °C before each subsample was analysed as described above.

### Chemical analysis

Samples were analysed for the PCBs congeners 28, 52, 101, 118, 138, 153, 170, 180, 194; IUPAC notation from Ballschmiter and Zell (1980). Among the 9 PCB compounds investigated in this study, the CB 28, 52, 101, 118, 138, 153, 180 were selected by the Commission of the European Communities (BCR) (Griepink et al. 1990). These compounds are considered as tracers of PCBs pollution and are the major constituents in DP6 (equivalent to Aroclor 1260), which is the industrial component widely used in France.

Usually, to estimate the total PCB, we use as formulae: tPCB = (CB118 + CB138 + CB153 + CB180) × 100/41 (Perez et al. 2003; Wafo et al. 2006, 2012).

In this study, we considered total PCBs as being the sum of the analyzed congeners. With this approach, we intended to facilitate the interpretation of the results, being aware that this does not provide an accurate calculation of total 209 congeners. By using the sum of the analyzed congeners, we consider that we get closer to the reality.

#### Sample extraction and quantification

All the samples were lyophilised, sieved through a 2 mm sieve, and then stored in amber glass bottles at −18 °C until analysis. Prior to analysis, the water content of each sample was determined systematically on a 1 g subsample dried at 105 °C for 24 h. The value obtained (%) is added to the results for each PCBs congener. 2 g of sediment was extracted using Pestipur^®^ hexane in a soxhlet apparatus. The resulting extracts were purified with concentrated sulfuric acid according to Murphy (1972), and then desulfurized with TBA-Sulfite Reagent (Jensen et al. 1977). Liquid chromatography was performed on these extracts using silica gel and alumina column, following previously described procedures (Wells et al. 1985). The resulting extract was concentrated to 1 mL. Analyses were performed with a HP 6890 series gas chromatograph equipped with a 63Ni electron capture detector (ECD) as described previously (Perez et al. 2003; Wafo et al. 2006, 2012).

#### Quality control

Glassware was cleaned before use with the detergent TFD4 dec FT30, dried at 200 °C for at least 24 h, and rinsed at least twice with the solvent (hexane) before use. All traces of organochlorinated compounds were removed from the extraction cartridges (22 × 80 mm, no. 350211, Schleicher & Schull) by conducting a blank pre-extraction for 12 h under normal conditions. For each series of extracts, a blank, certified sample (BCR-536) was used to provide an internal validation. For this certified sample, congeners 28, 52, 101, 118, 138, 153, 180 were analysed. Each sample was analysed in triplicate with recovery levels of 85–95 %. The detection limits for individual PCB congeners were 0.01 ng g^−1^ dry weights.

## Results and discussion

### Granulometry

Organochlorine compounds adsorbed on sediments can suffer degradation and desorption processes, the latter being strongly influenced by physical chemical factors of the sediment such as grain size or organic matter content (Bondi et al. 2006; Yang et al. 2011, 2012; Duan et al. 2013a). Results for grain size showed that fine fraction (F < 60 µm) was predominant over the entire bay. Sediment from Toulon Bay presented a high homogeneity, and a low variability with depth. This predominance of fine fraction will promote the accumulation of contaminants in the sediment.

**Fig. 2:**
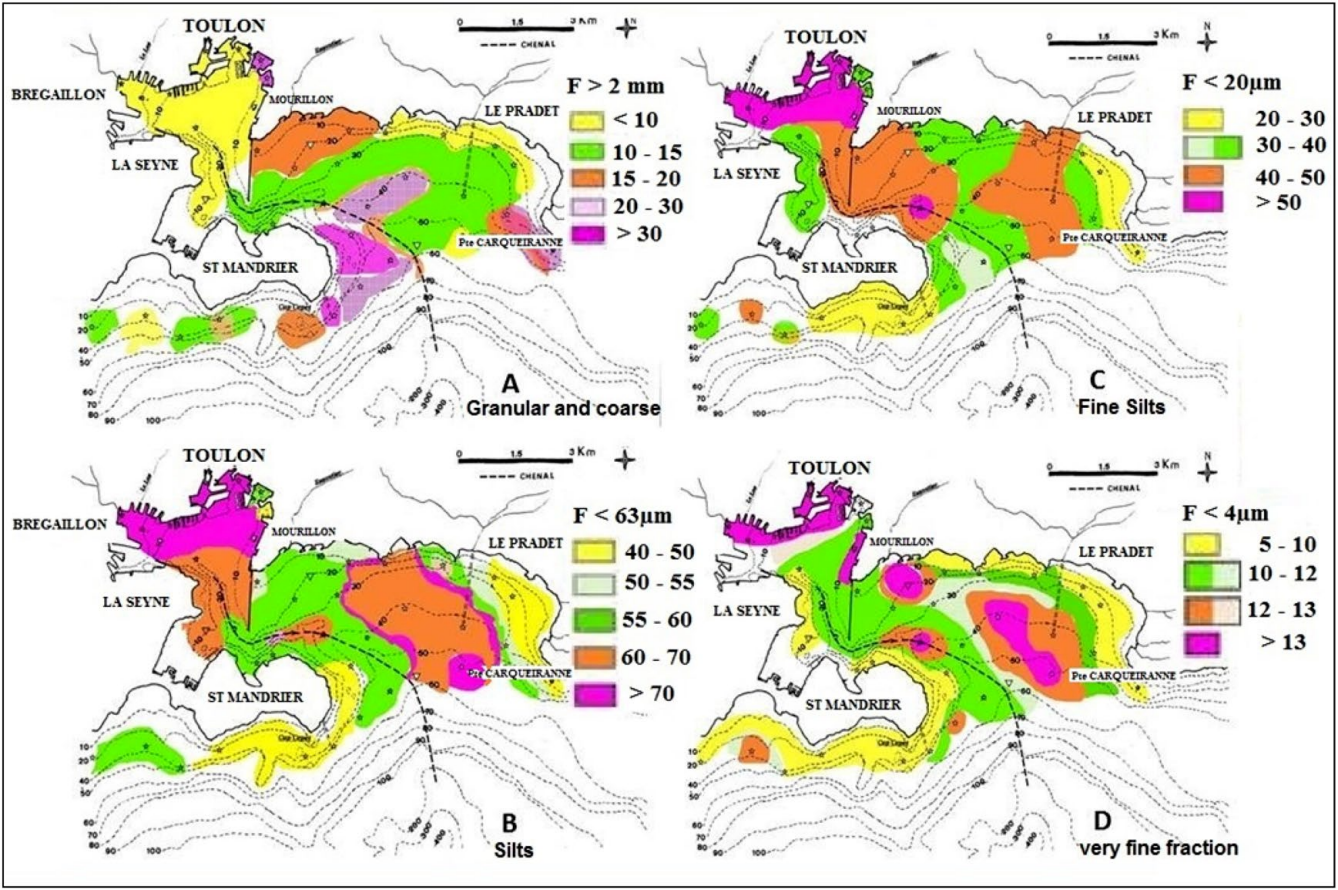
Spatial distribution of granule sizes (F>2m, F<63µm, F<20µm, F<4µm), with their percentages distribution (< 5 to > 30 %) in each superficial fraction sediments

The distribution of granule sizes was measured for layers 0–5 and 5–10 cm deep. For the sake of simplicity, since the differences between these two layers were usually small, an average value was calculated for each site. Figure 2a–d shows maps of the granule size distribution in total sediment for the most representative size ranges: greater than 2 mm (coarse sand and granules), less than 63 μm (silts), less than 20 μm (fine silts) and less than 4 μm (clays).

 Granular and coarse sands larger than 2 mm (Fig. 2a) corresponded mostly to gravel and debris from shellfish and vegetation. The proportion of these large granules varied from 2.6 to 43.9 %, reflecting the diversity of the sites studied. Silts smaller than 63 μm (Fig. 2b), mostly reflecting organic pollution (Taylor and Boult 2007), were detected at levels ranging between 83 and 45.3 %. The levels of 60–70 % were found over the remainder of the zones tested extending into the larger bay. The distribution of fine silts smaller than 20 μm was very similar to that of coarser silts (Fig. 2c). The major difference was detected in western regions of the larger, external bay where levels of 42–51 % may reflect an increased amount of very fine (F < 4 μm) rather than moderately fine sedimentation. This very fine sediment fraction (F < 4 μm) corresponds to clay-like particles, which are likely to be a better absorbent. This fraction was preferentially located in eastern regions of the larger, external bay (Fig. 2d).

In sum, the granulometric analysis reveals different behaviours, which vary depending on the geographical location. The zones containing the highest levels of fine sediment (70–80 %) are located in the most enclosed part of the bay in the north, where various activities are concentrated. The absence of tides in the Mediterranean, along with the presence of the pier, mitigates the impact of ocean currents. Waters renew slowly in these zones, which is favourable to the accumulation of fine particulate matter. Additional file 1: Table S1 shows the mean composition of six majors’ granules sizes (lower than 4, 4–20, 20–63, 63–200, 200–2000 and larger than 2000 μm) from each 2 cm layer.

Sedimentation and confinement in the smaller, internal bay tended to favour homogenous granule sizes, except for sand and particles larger than 2 mm, whose presence was most evident at sampling site 15. Sampling sites 23 and 52 in the larger external bay exhibited very different profiles. Site 23 was rather shallow and received the efflux from the Eygoutier River. Large granules were highly represented: 16.1 % for sands of 200–2000 μm and 13.4 % for coarse sands up to 2 mm. Site 52, the deepest (58 m deep), was characterised by important silting, with granules smaller than 63 μm (78 %) associated with a fraction of large granules (F > 2 mm) (8.1 %). The sandy granule fraction (200–2000 μm) was very low (1.3 %). This reflects the dual origin of granules in this rather heterogeneous area.

### PCB levels in superficial sediments

In the 0–5 and 5–10 cm layers from all parts of the bay, the total PCBs levels varied from 1.8 to 1869 ng g^−1^ and from 1.6 to 3188 ng g^−1^, respectively. As shown in Additional file 2: Figure S3A, the PCBs levels in the 0–5 cm layer varied from maxima north of the smaller bay (1869 ng g^−1^) to minima east of the larger, external bay (2.2 ng g^−1^).

PCBs concentrations higher than 100 ng g^−1^ were restricted to zones of port activity north of the small bay and very locally at one sample site (20) in the south. The spread of PCBs pollution to the south of the small bay and its opening into the larger bay follows currents flowing around the St Mandrier peninsula. This spread could be traced along the sampling sites 15, 18, 25, 26, 40 and 41, where PCBs levels were in the range 34–74 ng g^−1^. PCBs levels fell steadily from 13 to 2 ng g^−1^ with the distance north and east of the larger bay as well as towards the Anse des Sablettes.

The distribution of PCBs in samples of the deeper 5–10 cm layer (Additional file 2: Figure S3B) is similar to that in the upper 0–5 cm layer, especially in the smaller bay. In the larger bay, regions of very low PCBs levels extended over wider zones from the shore of Mourillon to the Carqueiranne point in the east. We note that the high PCBs level, 210 ng g^−1^, measured at sample site 41 in the southern region of the larger bay, indicates potential accumulation in the coastal sector.

Additional file 3: Figure S4 compares the PCBs levels measured in the upper (0–5 cm) and lower (5–10 cm) layers. The concentration ratios show that in the smaller bay there has been a quasi-general decrease in PCBs, except in sites 6 and CS, while in the larger bay a balance between PCB influx and removal may exist. These phenomena may be linked to sediment disturbance in this zone due to maritime traffic and dredging operations.

**Fig. 3:**
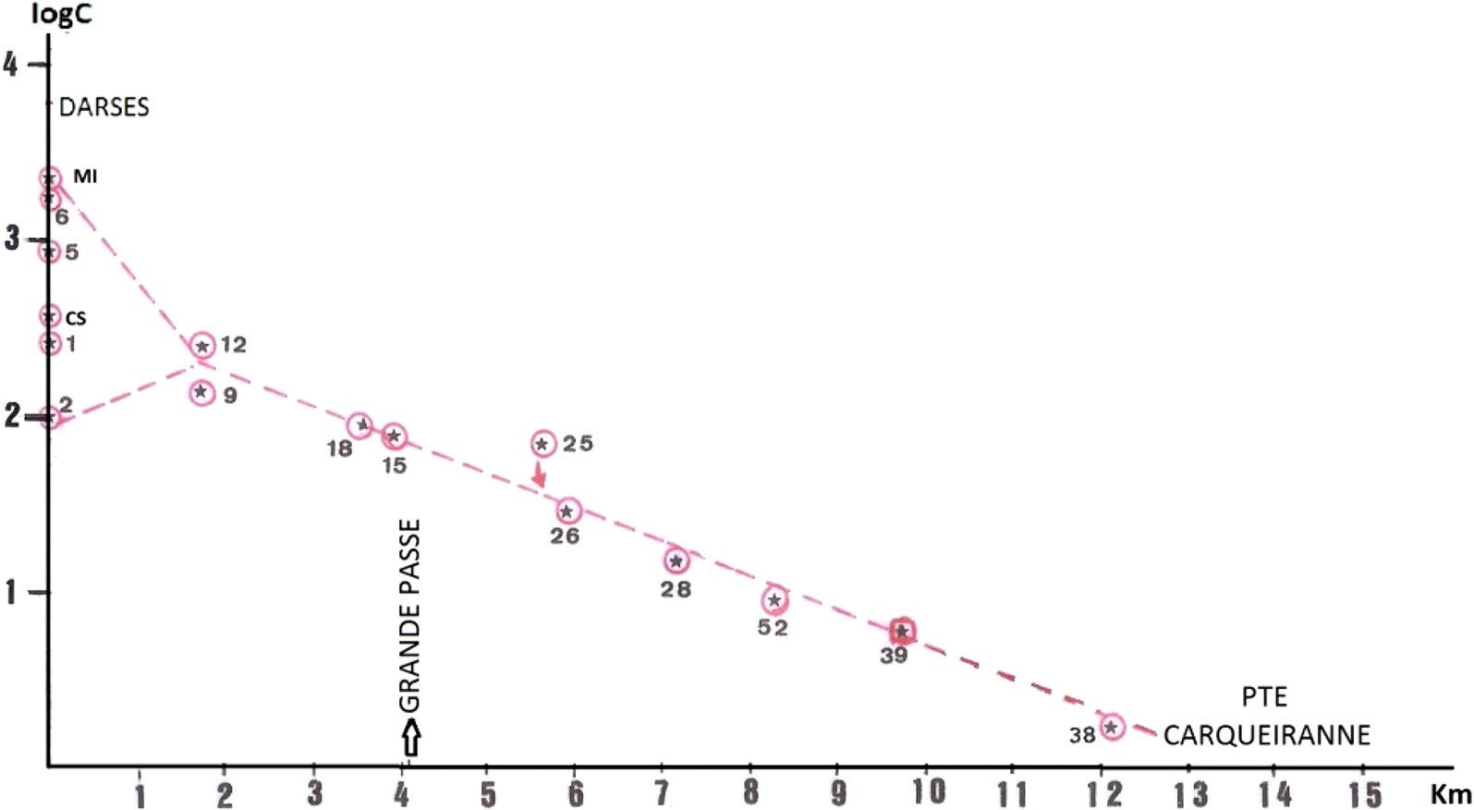
Concentration profile of total PCBs between the small bay (Station 1 to 18) and the large bay (Stations 25 to 52)

In Fig. 3, PCBs level variations between the small and the large bays are plotted as a logarithm of total PCBs concentration. This graph is based on data from a series of sampling sites located between the dock zones in the north of the small bay to the point of Carqueiranne. A good fit indicates the regular pattern of PCBs dispersal.

At a given sampling site, the fate of PCBs in shallow sediments depends on the transfer and sedimentation of particulate matter from a coastal site of origin to the site of accretion and on the stability of the conditions at the sampling site. We estimated relative proportions based on different homolog groups of analysed PCBs. Using Phenochlor DP6 as an example; we distinguished four homolog groups of congeners (3 + 4, 5, 6, 7 Cl). Assuming that dechlorination should involve first the most heavily chlorinated congeners (Brown et al. 1987; Wafo 1996; Fang et al. 2013; Demirtepe et al. 2015), we classified sites according to a decreasing order of heptachloride compounds. The percentages of other congener groups might vary according to the kinetics of dechlorination at each site.

Variation curves were generated for PCBs with 3, 4 or 5 Cl and with 7 Cl in order to illustrate changes in the sampling sites, using heptachlorinated PCBs as a reference. The degree of dechlorination assessed as an index of PCBs degradation was not identical at all sites, but the variations demonstrated a general trend that was similar for both layers (Additional file 4: Figure S6).

### The French Legislation level for PCB admitted for dredging waste

**Fig. 4:**
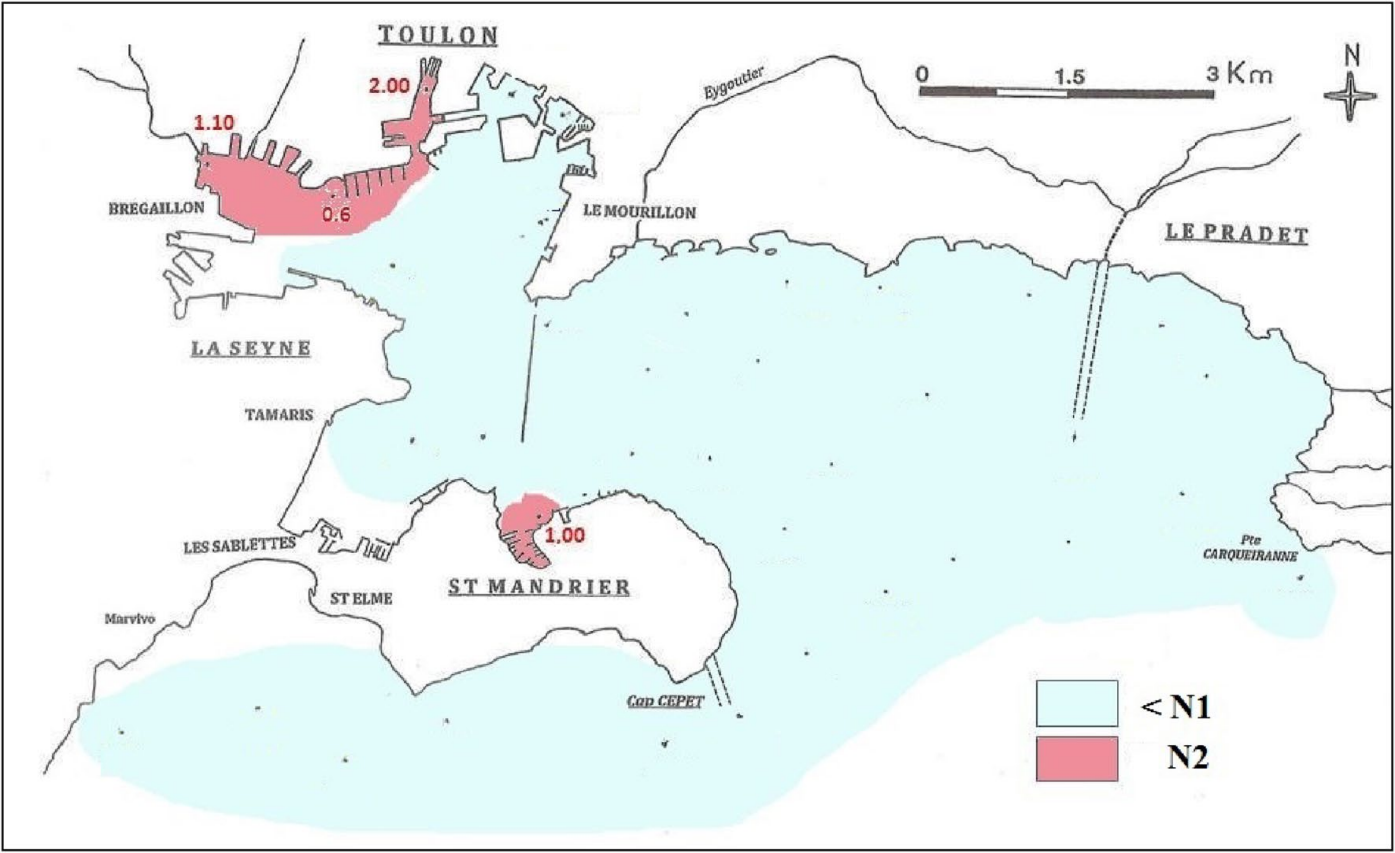
Spatial distribution of the levels of pollution compared with target values

 The current French legislation on dredging waste is based on multiple guidelines (Alzieu 2005) for the level of pollutants that may be present in dockside zones. Values are expressed in mg kg^−1^ of dry sediment at two different levels: N1 (0.5 mg kg^−1^) and N2 (1.0 mg kg^−1^) for immersed sediments. At levels below N1, effects are thought to be neutral or negligible for the environment. Between levels N1 and N2, further toxicity assessments may be required depending on the project and on the extent to which N1 is exceeded. Above level N2, negative environmental effects are possible. Immersion may be forbidden and alternative solutions must be sought. Sediments in the bay may be estimated by averaging the values from the upper (0–5 cm) and lower (5–10 cm) layers at each sample site. Figure 4 shows those among the sampling sites that fall within the levels N1 and N2, as defined for PCBs.

PCBs pollution reaches N2 levels in a small proportion of the region considered, particularly at sample sites 5, 6 and MI north of the small bay and also around a localised source, St 20, south of the small dock. Except for these sites, PCBs pollution in Toulon Bay is below level N2. Heavy use of these molecules occurred during a relatively limited period. Legislation introduced in the 1970s has successfully suppressed PCBs manufacture and use (http://www.legifrance.gouv.fr) with positive effects on the marine environment around Toulon. For comparison, we note, however, that the level of PCBs pollution in aquatic sediment from the Marseilles bay has been measured at 1.6 mg kg^−1^, up to the N2 level (Wafo et al. 2006) and 0.75 mg kg^−1^, up to the N1 level (Agung Dhamar et al. 2012).

 The concentration ranges of PCBs in this study are compared to those reported from different locations of the Mediterranean region and other industrial or urban coastal environments (Table 1). It should be noted, however, that a quantitative comparison across reported PCBs compounds data is difficult because of variances in the time of sampling, specific congeners measured in each study, the sediment fraction analyzed, and the analytical methods used. With the exception of the small bay with high PCBs level (100–2530 ng g^−1^ dw); the PCBs concentrations in surface sediment in this study were also in the same order magnitude than those reported from other locations.

**Table 1:** Comparison of PCBs concentrations (ng g^−1^ dw) in the surface sediments of different coastal areas

Location	PCB	References
Lake Como, Italia	449.7–1672.1	Bettinetti et al. (2016)
Seine River Basin, France	2300	Lorgeoux et al. (2016)
Baltic	28	Sobek et al. (2015)
Northern France	126.8–194.4	Net et al. (2015)
Nadoor Lagoon, Marocco	2.5–20.5	Giuliani et al. (2015)
Rhône River, France	11.5–417.1	Mourier et al. (2014)
Port Elizabeth Harbour, South Africa	0.56–2.35	Kampire et al. (2015)
Qinzhou Bay, South China	1.62–62.63	Zhang et al. (2014)
Monastir Bay (Tunisia, Central Mediterranean)	3.1–9.3	Nouira et al. (2013)
Mediterranean coastal environment, Egypt	0.29 – 288	Barakat et al. (2013)
Egyptian Mediterranean coast	7.06–75.17	Aly Salem et al. (2013)
Eastern Aegean Coast, Turkey	26.07	Kucuksezgin and Gonul ( 2012)
Rhône River, France	78–281	Desmet et al. (2012)
Abu Qir Bay (Egypt)	45	Khairy et al. (2012)
Cortiou, Marseille, France	750	Agung Dhamar et al. (2012)
Port of Spain	62–601	Mohammed et al. (2011)
Mar Piccolo, Taranto Ionian Sea, South Italy	2–1684	Cardellicchio et al. (2007)
Naples harbor, Italy	10–899	Sprovieri et al. (2007)
Cortiou, Marseille, France	12.68–1559.3	Wafo et al. (2006)
Dalian Bay	58.1 (average)	Xing et al. (2005)
Singaore’s coast	1.4–329.6	Wurl and Obbard (2005)
Narragansett Bay, USA	20.8–1760	Hartmann et al. (2004)
Masan Bay, Korea	1.24–41.4	Hong et al. (2003)
Alexandria harbor, Egypt	0.9–1211	Barakat et al. (2002)
Daya Bay, China	0.85–27.37	Zhou et al. (2001)
Hong Kong’s coast	3.5–25.1	Hong et al. (1999)
Victoria harbor, Hong Kong	3.2–27	Connell et al. (1998)
Coastal Barcelona offshore	4.0–64	Tolosa et al. (1995)
Abu-Quir Bay, Egypt	53–231	Abdallah and Abbas (1994)

### Establishment of reference values to estimate PCBs pollution

PCBs are ubiquitous in environmental samples. Since they do not occur naturally but are produced industrially, their maritime reference levels should be near zero, given their global dispersion. Clearly this is unrealistic. In this context, it is difficult to define a range of concentrations of total PCBs that properly reflects the gradients from the coast to the open sea. Levels of PCBs in the open sea might be used as “normal” minimal concentrations.

For sediments of a relatively confined environment such as the Western Mediterranean Sea, this value could be deduced from previously measured concentration gradients in our laboratory from 1995 to 2009 (Perez et al. 2003; Wafo et al. 2006). To this end, we present a range of concentrations inferred statistically from the analysis of over 1500 samples using similar methodologies. Sediments were collected in the North-Western Mediterranean from coastal regions to depths of down to 2000 m between Corsica and the Balearic Islands. The results are expressed as tPCBs, with five concentrations levels (0–10–25–50–100 ng g^−1^ dw) used to represent transitions between non-polluted and heavily polluted sediments (Table 2).

**Table 2:** Proposed reference values

tPCBs (ng g^−1^ dw)	Sediment qualification
<10	Not polluted
10–25	Slightly polluted
25–50	Moderately polluted
50–100	Polluted
>100	Very polluted

We note that these data were obtained from the open sea and so exclude very high PCBs levels in harbour deposits, which should be considered as industrial wastes.

### Using the core samples to determine PCBs distribution in time

#### Alkane distributions in a core sample

**Fig. 5:**
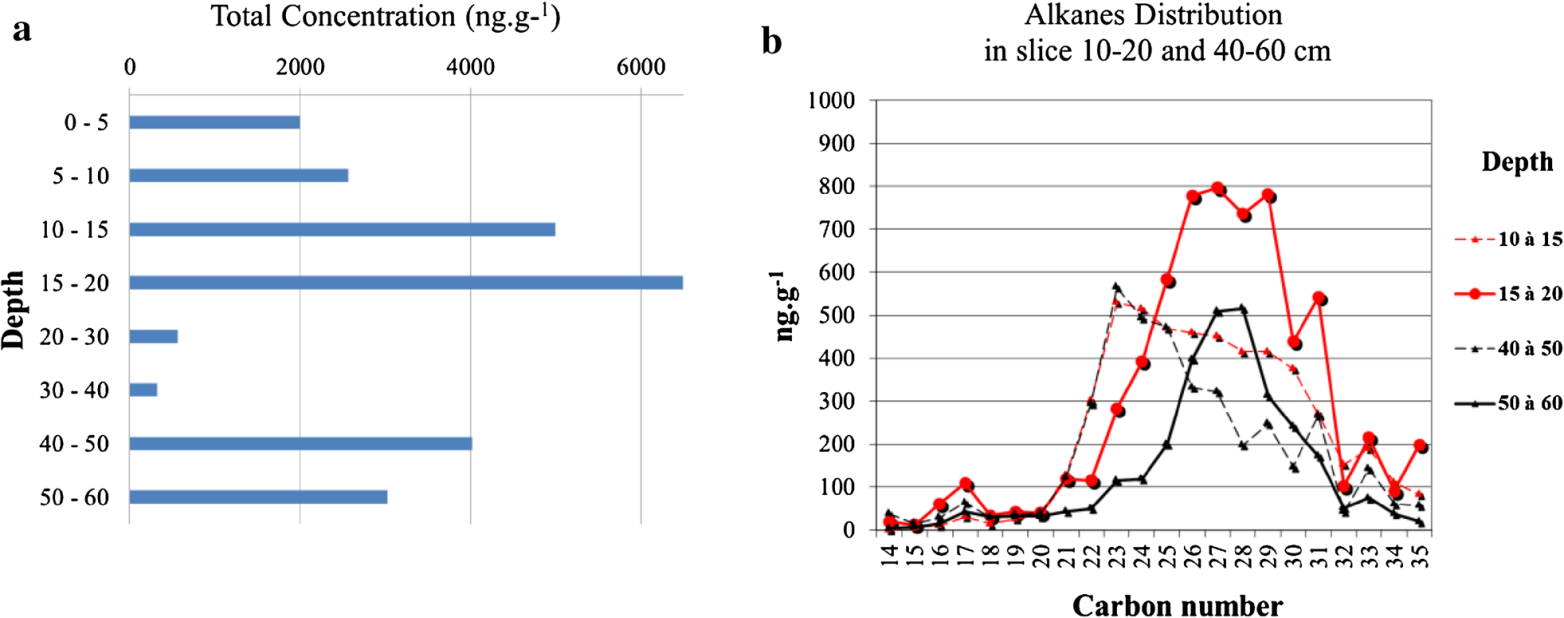
Depth profile *n*-alkane distribution by slice, from 10 to 20 and 40 to 60 cm

Alkanes are saturated chain hydrocarbons. They may be natural, anthropogenic, pyrolytic or petroleum-related. These categories may reflect the number of carbon atoms. A natural origin for specific alkanes is indicated by chains with odd numbers of carbon atoms. The C_17_ alkane has been linked to a marine phytoplanktonic origin (Aboul-Kassim and Simoneit 1995, 1996; Colombo et al. 1989, 2006), while C_27_, C_29_, C_31_ and C_33_ alkanes have a terrestrial origin, possibly related to petroleum (Mille et al. 1981; Mahler et al. 2005; Yunker et al. 2002, 2015; Foster et al. 2015). A study of alkanes, reported by Milano (1990), was made using a 60 cm deep sample obtained from a site very close to sample site 15 of this study. We use data from this analysis to estimate an average sedimentation rate of PCBs in the cores for our study. Detailed results from this analysis, based on variations in alkanes C_14_ to C_36_ at each level of the sample, show the distribution of alkanes C_20_ to C_32_ at two characteristic levels along the core, revealing an overall increase in the total concentration (10–20 and 40–60 cm) (Fig. 5a).

In each case, a large and rather symmetric peak between C_21_ and C_32_ with a maximum at C_27_ is followed by a smaller, asymmetric peak displaced towards C_23_ (Fig. 5b). We can make a reasonable hypothesis on the origins of these variations. The scuttling of the French Mediterranean fleet on the 27th of November 1942 (Antier 1992) is the only recent event that might have initiated such a massive release of hydrocarbons. One hundred and seventeen ships were sunk, and all naval dockyard facilities destroyed. While the sabotage operation lasted for 2 h, fires and explosions continued for several days. Huge quantities of toxic waste released from the destroyed ships, docks, buildings, munitions and fuel were added to the harbour sediments. This massive and rapid pollution of the entire Toulon Bay was followed by a long period of recovery which prolonged, even though more moderately, the contamination of the bay. The 1942 cataclysm also has biological correlates in the sediment record. The level (20–30 cm) corresponding to the scuttling of the fleet is associated with a transition from grey, clay *Posidonia* beds to grey beds of decaying *Posidonia* debris, testimony to a strong perturbation of the underwater biological habitat.

Following our hypothesis, the paroxysmal phase should be attributed to 15–20 cm layers. High levels of pollution seem likely to have affected larger invertebrates, consequently reducing the sediment disturbance that they generate. In contrast, an influx of novel PCBs would tend to facilitate re-arrangements that would transfer previously accreted PCBs to higher levels in the sediment structure. At the same time, hydrocarbon chains should degrade, leading to a new distribution with shorter chains and a shift of the peak to C_23._

These observations allowed us to date the 15 cm layer back to 1942. Average sedimentation could then be estimated at 0.32 cm per year. This provided a valuable basis for studying the chronology of sediment pollution in the smaller bay.

Usually, sediment cores have been used to estimate historical deposition and trends of persistent environmental contaminants, whereby the chronology of contamination is linked to the measurement of ^210^Pb or ^137^Cs activities in the sediment layers as a function of depth and time (Hites 2004; Covaci et al. 2005; Stern et al. 2005). The pioneering study by Hites et al. (1977) on the historical record of sedimentary PAHs ushered in the use of sediment cores to trace the history of accumulation of many pollutants, including PCBs (Boonyatumanond et al. 2007; Zennegg et al. 2007) in many aquatic ecosystems around the world. Sediment can be seen as a geologic history or memory that retains patterns from the past. The Toulon harbour has experienced a series of events that are likely to have played a role in affecting the sediment at different dates. We can attempt to identify the traces of these events to estimate the sedimentation speed at different sites. Using the ^210^Pb or ^137^Cs activities, (Tessier et al. 2011) found 0.27 cm as an average sedimentation for the same core in the small bay. The results obtains in our study sediments are similar to those found elsewhere such as Masan Bay, Korea (Hong et al. 2010), Lake Maggiore, Italy (Guzzella et al. 2008), the Norwegian Arctic (Evenset et al. 2007), the Strait of Georgia, Canada (Johannessen et al. 2008), Thailand (Kwan et al. 2014), Haizhou Bay in China (Xing et al. 2005; Zhang et al. 2014), and Guaratuba Bay (Combi et al. 2013). Similar sediment PCBs profiles were observed in Changjiang Estuary and adjacent East China Sea (Yang et al. 2012; Duan et al. 2013a); In The Seine River Basin-France (Lorgeoux et al. 2016, The Seine River Basin, Paris), Nador Lagoon-Morocco(Giuliani et al. 2015), and The Rhône River-France (Mourier et al.^2014^; Desmet et al. 2012).

#### PCBs distribution in core samples

Minimal and maximal concentrations from each sample are presented in Table 3.

**Table 3:** Minimum and maximum PCBs levels in the samples

Core	12		15		23		52	
	Min	Max	Min	Max	Min	Max	Min	Max
*tPCB (ng* *g* ^−*1*^ *)*								
Total sediment	1.9	653	0.8	96	1	14.8	1.3	11.3
(F < 63 μm)	3	739	1	140	2	24	2	13

**Fig. 6:**
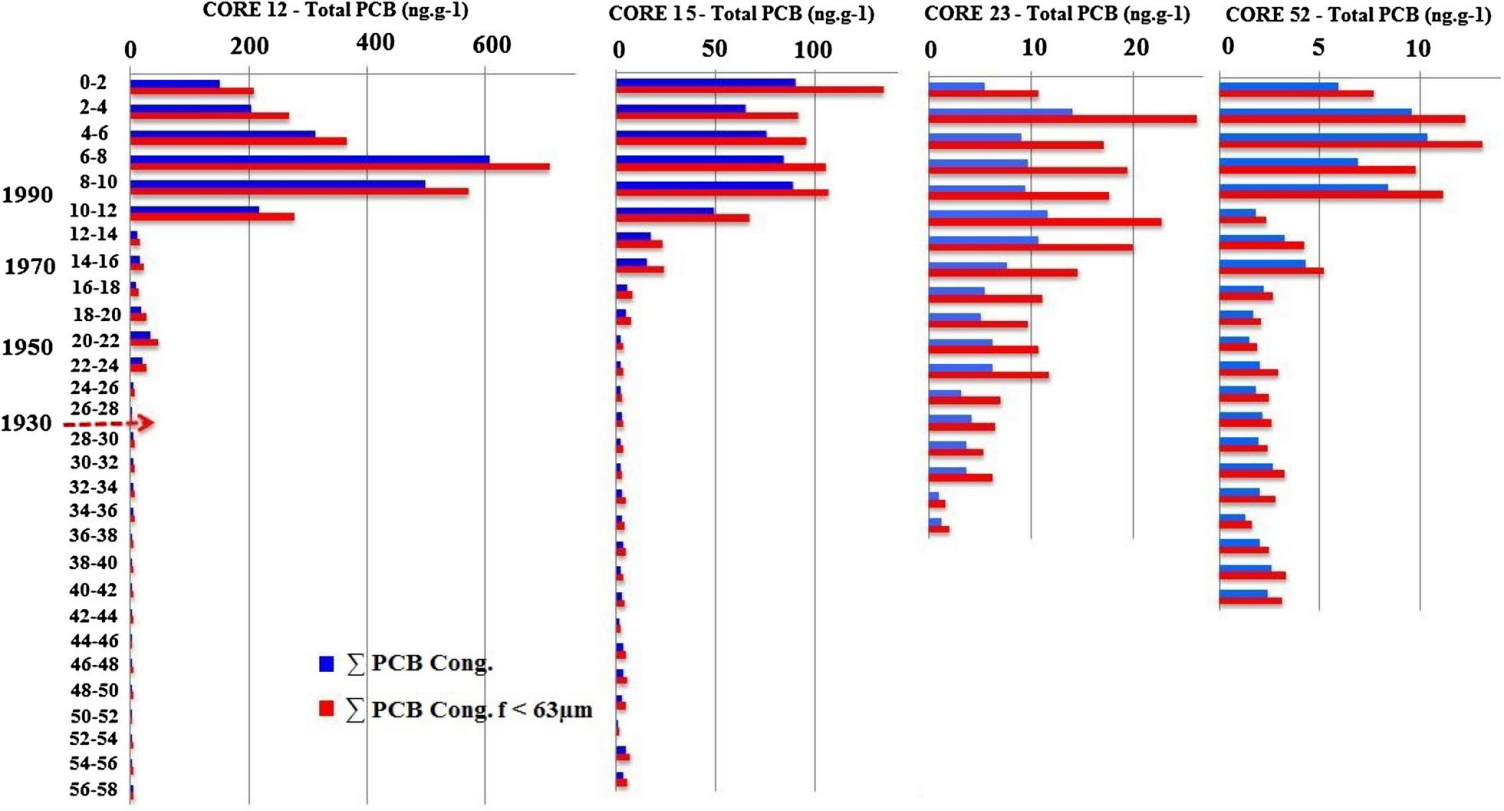
Depth profiles of PCBs levels in the samples

As shown in Table 4, PCBs levels, both in total sediment and in the fine fraction (F < 63 µm), were highest in samples from site 12. Comparison of samples from different sites (Fig. 6) revealed that these differences appeared starting at depths of 26 cm, corresponding approximately to the 1930s period. PCBs levels lower than 100 ng g^−1^ at depths down to 12 cm revealed lower rates of PCBs accretion. The maximum concentrations during this period were similar to the 1940–1950 period concentrations. A rapid, massive increase in PCBs content started in the 1960s and 70s, with levels reaching a maximum of 650 ng g^−1^ at depths of 6–8 cm in the 1980s–1990s. PCBs levels then declined regularly to values near 200 ng g^−1^ at the surface, which corresponds to today’s values.

Part of the reduction in PCBs levels can be attributed to the legislation of the 1970s prohibiting their sale and use. Another factor may also have influenced PCBs pollution in Toulon Bay: in the 1970s, naval submarines were replaced by civil dockyard activities at Mourillon. Differences in PCBs levels between the total sediment and its fine fraction were constant and rather small, as expected from the nature of deposits in this zone of the bay.

In the sample core 15, PCBs were first detected at depths of about 26 cm, corresponding approximately to deposits from 1930. PCBs levels increased after the war (1945), especially from 12 cm up to the surface (1970–1980), where PCBs exceeded 50 ng g^−1^. Maximal levels of 140 ng g^−1^ were found in the fine fraction (F < 63 µm) at the surface. The sample core 12 differs in several aspects from sample 15. First, there was an increased mean ratio between PCBs levels in the fine fraction and in total sediment (1.32 in sample 12 and 1.55 in sample 15). This correlates with the larger sediment granule sizes in samples from site 15. Second, maximal PCBs levels were higher, with a ratio of 3.7 for mean levels in the most polluted part of the respective samples. Third, the depth profiles of PCBs levels differed. A peak could be clearly observed for samples at depths of 6–8 cm, while in samples from site 15, PCBs levels fluctuated but remained elevated up to the surface. The sample core 23 is situated far from pollutants originating from the industrialised zones of Toulon. In the sample core 52 the PCB content was very low (maximum of 13 ng g^−1^). PCB levels did not exceed the background (5 ng g^−1^) at levels lower that 16–18 cm. Sediment from this site was largely clay-like with 85 % of F < 63 μm in the most recently deposited sediment.

There is a very evident decrease of PCBs concentrations in the late 1990s, correlated with the legal ban in 1986 in France (http://www.legifrance.gouv.fr). The bar graphs of specific PCBs depth distributions shown in Additional file 5: Figure S10 demonstrate a clear division above and below 12 cm for different samples. From the surface to a depth of 12 cm, different congeners follow stable, equilibrated distribution with no dominant contribution. In contrast, at depths below 12 cm, the three least chlorinated congeners (CB28, 52, 101) dominate, with maximal levels at 16 and 30 cm separated by a minimum at 20–22 cm.

Differences between upper and lower sample layers may be interpreted as resulting from increases in PCBs pollution, but also as the effect of lower PCBs levels during periods including the Second World War and its aftermath. Reduced PCBs efflux probably encourages a more complete biodegradation and so enhances the proportion of less-chlorinated PCBs.

In the sample core 15, except for an ‘abnormal’ peak corresponding to the 8–10 cm layer, 7-Cl PCBs levels fell steadily from the surface to deeper layers (from 39 to 10 %). The level of 3–4 Cl PCBs increased proportionally (from 1 to 19 %) and so did the level of 5-Cl PCBs (from 8 to 29 %). The level of 6-Cl PCBs remained rather constant (41–47 %). At depths below 10 cm, few changes were evident, and 3–4 Cl PCBs were practically absent. We suggest that PCBs degradation starts in the less oxygenated regions of the sampled sediment and then accelerates logarithmically. These processes occur more slowly at lower depths (including the 20–25 cm layer) where PCBs levels are very low, close to baseline noise. In the sample core 23, a regular sequence of changes in different PCBs components was evident between the surface and a depth of 28 cm for this sample. The level of 7-Cl PCBs fell from 37 to 7 %, while 3 + 4 + 5Cl PCBs increased from 7 to 41 %. The level of 3-Cl PCBs was relatively high, in sharp contrast to samples from sites 12 and 15, where they are essentially absent from near-surface depths. PCBs degradation apparently starts close to the efflux from the Eygoutier River. These data are somewhat less clear than for samples from other sites. Presumably they result from the more heterogeneous granule structure of this sample, which could favour more uniform PCBs degradation processes. Additional file 6: Figure S11 summarises the tendency of changes in PCBs chlorination in our samples.

For sample core 52, with the exception of the 8–10 cm layer, which was characterised by reduced levels of 7-Cl PCBs in favour of 5-Cl PCBs, the degradation process manifested in a decrease in 7-Cl PCBs levels that was compensated for by and increase in 3 + 4 + 5-Cl PCBs levels (Additional file 6: Figure S11). This process is comparable to the one observed in the other samples. Despite being linked to a time factor, and consequently to a sedimentation speed that is not identical to that of the other samples, the differences in slope can be observed between the different sites. We should stress here that low, residual levels may not depend exclusively on degradation processes. The levels of less chlorinated PCBs that are more soluble and have lower molecular weights may also result from the expulsion of these derivatives in water columns.

## Conclusion

PCBs are a permanent contaminant of coastal sediments, but are limited quantitatively and qualitatively over time. In the present study, we showed that PCBs pollution was limited in the small Toulon Bay, a relatively protected harbour zone. The PCBs content reached and even exceeded average levels of 2530 ng g^−1^ (level N2) at certain sites. In the larger, more open bay, the PCBs content was lower, between 1.9 and 136 ng g^−1^ on average (level N1) indicating that that the risk posed by PCBs is limited. The PCBs concentrations in surface sediment in this study were also in the same order magnitude than those reported from other locations.

It can be hoped that, after the 1970s legislation on PCBs fabrication, sale and use, new polychlorinated biphenyl pollution is at an end. The PCBs concentrations in surface sediment in this study, especially in the larger bay, were also in the same order magnitude than those reported from other locations.

Our study resolved levels of PCBs contamination in total sediment and especially in sediment fractions of silts and clays (<63 µm). A relatively uniform PCBs degradation was estimated for both bays studied based on the analysis of the fractions of PCBs congeners with different numbers of Cl atoms.

Five ranges of PCBs levels are proposed for a rational definition of sedimentary pollution at other sites. Dated sediment core samples were able to capture the historical deposition of PCBs in Toulon Bay reflecting important events and factors that influenced the deposition of these compounds. The sedimentation rate was estimated from the timing of major events which left an imprint on the geological memory. These events included the traces of military conflicts, the increase in PCBs use in the 1950s and 1960s and changes in the course of a river flowing into the bay. In this way, we could follow the PCBs pollution history over about 80 years and estimate a sedimentation rate of about 0.32 cm year^−1^ in the small Bay of Toulon.

## Additional files

10.1186/s40064-016-2715-2 Means and S.D. of grain sizes in core sediments.
10.1186/s40064-016-2715-2 Spatial distribution of PCBs levels across both bays (A: 0–5 cm layer; B: 5–10 cm layer).
10.1186/s40064-016-2715-2 Total PCBs ratio: 0–5 cm layer versus 5–10 cm layer.
10.1186/s40064-016-2715-2 Trends in the proportion of 3 + 4 + 5 Cl and 7 Cl across sampling sites.
10.1186/s40064-016-2715-2 Contributions of specific PCBs to the total PCB load from sample sites.
10.1186/s40064-016-2715-2 Depth profiles of 3 + 4 + 5 Cl and 7 Cl-PCBs in samples.
