# Supplementary material for: A chronicle of the changes undergone by a maritime territory, the Bay of Toulon (Var Coast, France), and their consequences on PCB contamination
Source: Springerplus. 2016 Aug 2;5(1):1230. doi: 10.1186/s40064-016-2715-2 (PMC4970988; doi:10.1186/s40064-016-2715-2)
Supplement: Supplementary file 1 — 10.1186/s40064-016-2715-2 Means and S.D. of grain sizes in core sediments. [file 40064_2016_2715_MOESM1_ESM.docx]

**Table S1:** Means and S.D. of grain sizes in core sediments

| **Core** | **Fraction (µm)** | | | | | |
| --- | --- | --- | --- | --- | --- | --- |
|  | **< 4** | **4 - 20** | **20 - 63** | **63 - 200** | **200 - 2000** | **> 2000** |
| **12** | 11.8 ± 1.9 | 35.1 ± 4.3 | 25.5 ± 1.8 | 16.0 ± 2.9 | 7.0 ± 3.2 | 4.6 ± 2.7 |
| **15** | 9.9 ± 1.6 | 34.1 ± 3.7 | 24.0 ± 2.0 | 15.8 ± 2.4 | 7.4 ± 4.0 | 9.1 ± 2.8 |
| **23** | 7.8 ± 1.2 | 27.7 ± 3.4 | 19.6 ± 1.9 | 15.5 ± 2.8 | 16.1 ± 5.3 | 13.4 ± 3.6 |
| **52** | 16.8 ± 2.0 | 36.5 ± 2.9 | 24.6 ± 1.7 | 11.9 ± 3.3 | 1.3 ± 1.4 | 8.9 ± 3.6 |
