# Supplementary figures and images for: A chronicle of the changes undergone by a maritime territory, the Bay of Toulon (Var Coast, France), and their consequences on PCB contamination
Source: Springerplus. 2016 Aug 2;5(1):1230. doi: 10.1186/s40064-016-2715-2 (PMC4970988; doi:10.1186/s40064-016-2715-2)

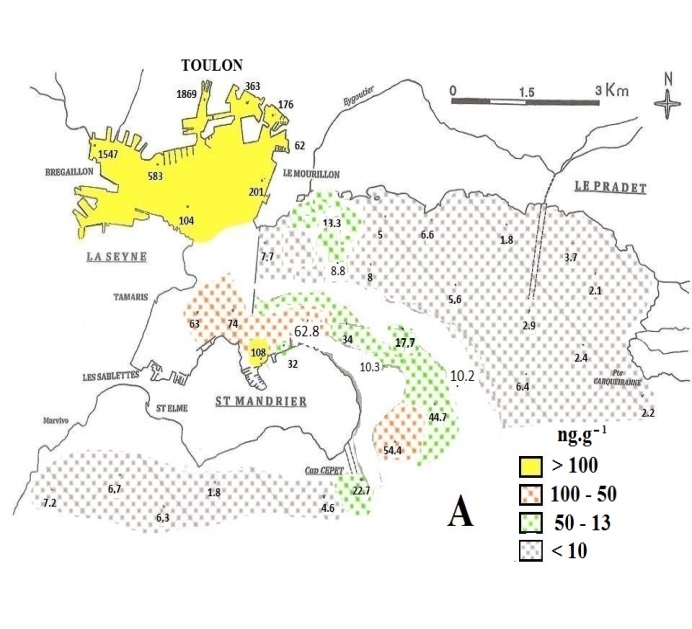

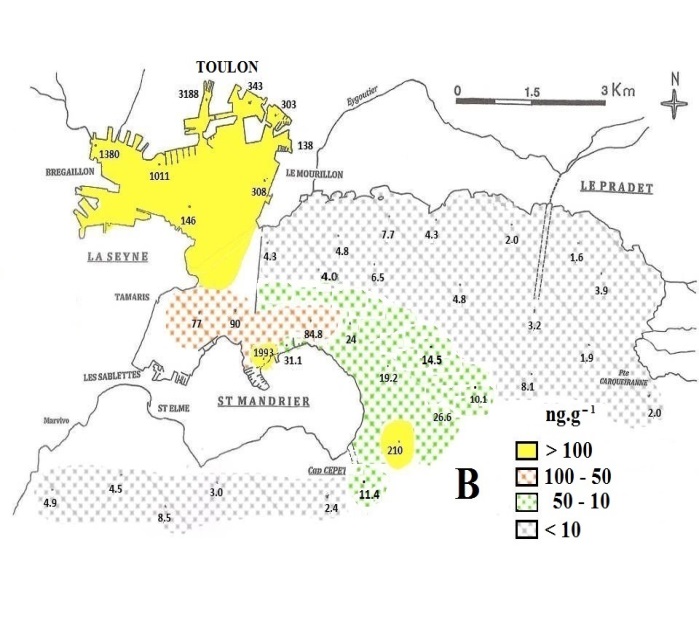


**Fig S3** Spatial distribution of PCBs levels across both bays (A: 0-5 cm layer; B: 5-10 cm layer)

Supplement: Supplementary file 2 — 10.1186/s40064-016-2715-2 Spatial distribution of PCBs levels across both bays (A: 0–5 cm layer; B: 5–10 cm layer). [file 40064_2016_2715_MOESM2_ESM.docx]

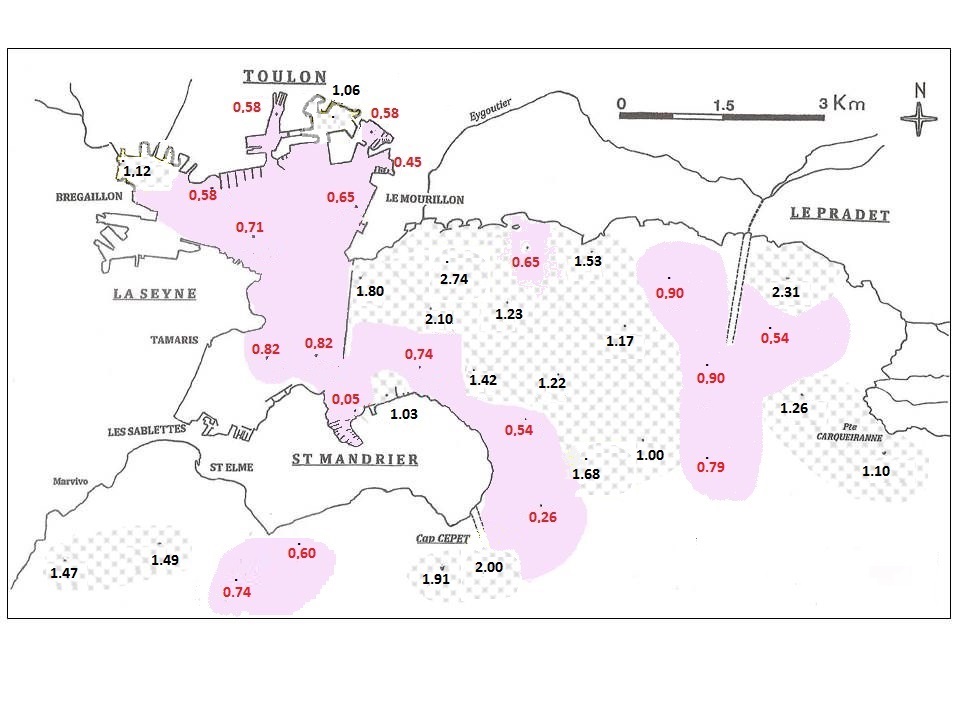


**Fig S4** Total PCBs ratio: 0-5 cm layer vs 5-10 cm layer

Supplement: Supplementary file 3 — 10.1186/s40064-016-2715-2 Total PCBs ratio: 0–5 cm layer versus 5–10 cm layer. [file 40064_2016_2715_MOESM3_ESM.docx]

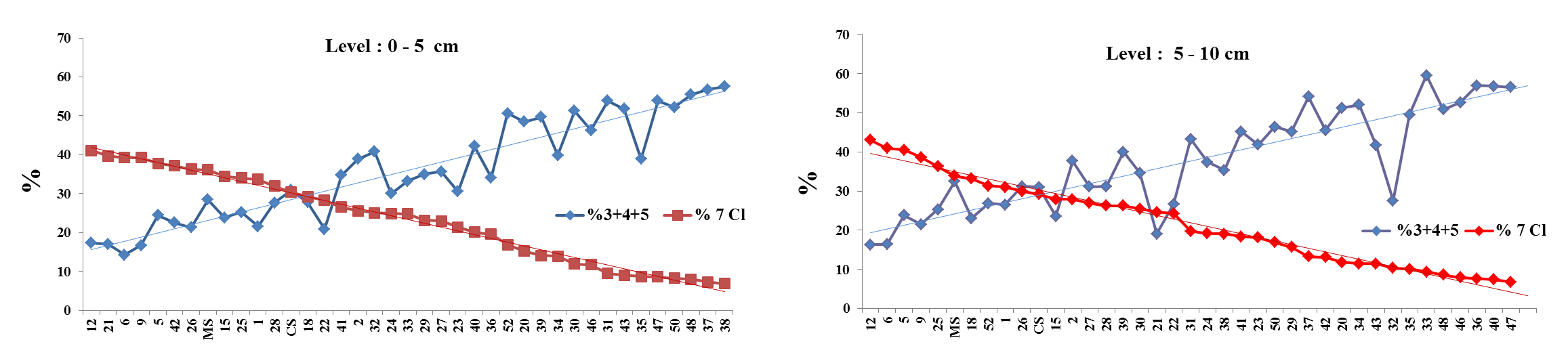


**Fig S6** Trends in the proportion of 3+4+5 Cl and 7 Cl across sampling sites

Supplement: Supplementary file 4 — 10.1186/s40064-016-2715-2 Trends in the proportion of 3 + 4 + 5 Cl and 7 Cl across sampling sites. [file 40064_2016_2715_MOESM4_ESM.docx]

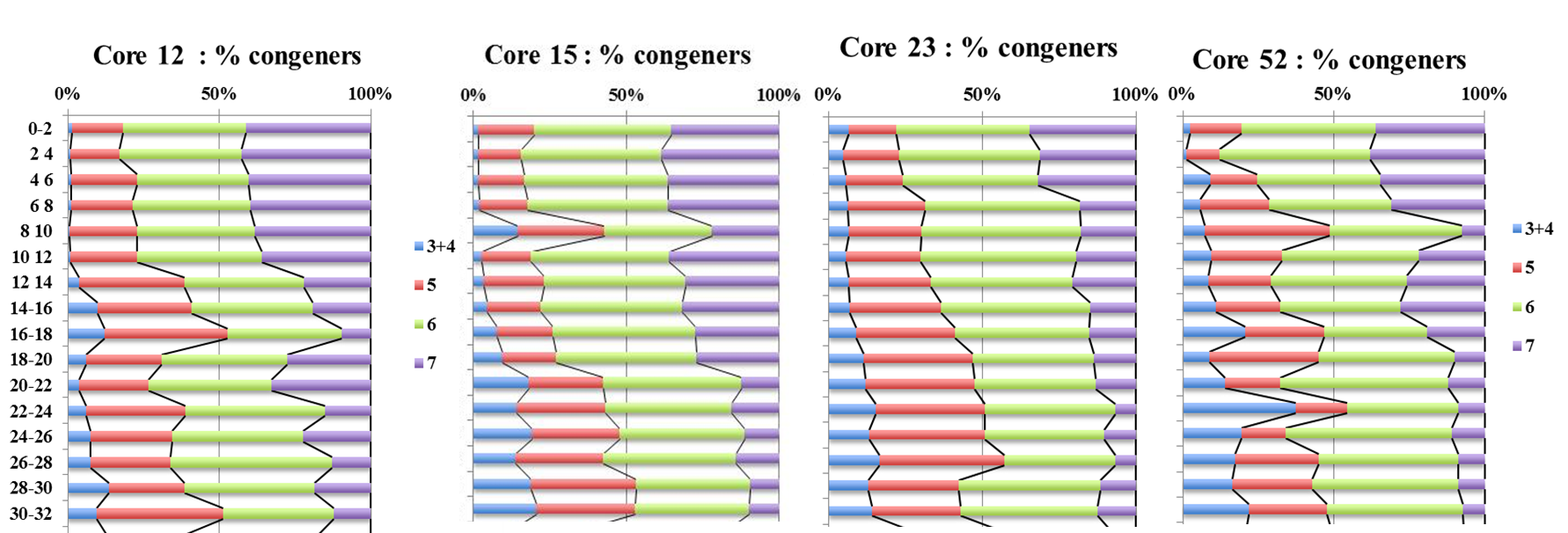


**Fig S10** Contributions of specific PCBs to the total PCB load from sample sites

Supplement: Supplementary file 5 — 10.1186/s40064-016-2715-2 Contributions of specific PCBs to the total PCB load from sample sites. [file 40064_2016_2715_MOESM5_ESM.docx]
