# Supplementary material for: A chronicle of the changes undergone by a maritime territory, the Bay of Toulon (Var Coast, France), and their consequences on PCB contamination
Source: Springerplus. 2016 Aug 2;5(1):1230. doi: 10.1186/s40064-016-2715-2 (PMC4970988; doi:10.1186/s40064-016-2715-2)
Supplement: Supplementary file 6 — 10.1186/s40064-016-2715-2 Depth profiles of 3 + 4 + 5 Cl and 7 Cl-PCBs in samples. [file 40064_2016_2715_MOESM6_ESM.docx]

**Fig S11** Depth profiles of 3+4+5 Cl and 7 Cl-PCBs in samples
